# Convolutional neural networks: an overview and application in radiology

**DOI:** 10.1007/s13244-018-0639-9

**Published:** 2018-06-22

**Authors:** Rikiya Yamashita, Mizuho Nishio, Richard Kinh Gian Do, Kaori Togashi

**Affiliations:** 1 Department of Diagnostic Imaging and Nuclear Medicine, Kyoto University Graduate School of Medicine, 54 Kawahara-cho, Shogoin, Sakyo-ku, Kyoto, 606-8507 Japan; 2 Department of Radiology, Memorial Sloan Kettering Cancer Center, 1275 York Avenue, New York, NY 10065 USA; 3 Preemptive Medicine and Lifestyle Disease Research Center, Kyoto University Hospital, 53 Kawahara-cho, Shogoin, Sakyo-ku, Kyoto, 606-8507 Japan

**Keywords:** Machine learning, Deep learning, Convolutional neural network, Medical imaging, Radiology

## Abstract

**Abstract:**

Convolutional neural network (CNN), a class of artificial neural networks that has become dominant in various computer vision tasks, is attracting interest across a variety of domains, including radiology. CNN is designed to automatically and adaptively learn spatial hierarchies of features through backpropagation by using multiple building blocks, such as convolution layers, pooling layers, and fully connected layers. This review article offers a perspective on the basic concepts of CNN and its application to various radiological tasks, and discusses its challenges and future directions in the field of radiology. Two challenges in applying CNN to radiological tasks, small dataset and overfitting, will also be covered in this article, as well as techniques to minimize them. Being familiar with the concepts and advantages, as well as limitations, of CNN is essential to leverage its potential in diagnostic radiology, with the goal of augmenting the performance of radiologists and improving patient care.

**Key Points:**

*• Convolutional neural network is a class of deep learning methods which has become dominant in various computer vision tasks and is attracting interest across a variety of domains, including radiology.*

*• Convolutional neural network is composed of multiple building blocks, such as convolution layers, pooling layers, and fully connected layers, and is designed to automatically and adaptively learn spatial hierarchies of features through a backpropagation algorithm.*

*• Familiarity with the concepts and advantages, as well as limitations, of convolutional neural network is essential to leverage its potential to improve radiologist performance and, eventually, patient care.*

## Introduction

A tremendous interest in deep learning has emerged in recent years [1]. The most established algorithm among various deep learning models is convolutional neural network (CNN), a class of artificial neural networks that has been a dominant method in computer vision tasks since the astonishing results were shared on the object recognition competition known as the ImageNet Large Scale Visual Recognition Competition (ILSVRC) in 2012 [2, 3]. Medical research is no exception, as CNN has achieved expert-level performances in various fields. Gulshan et al. [4], Esteva et al. [5], and Ehteshami Bejnordi et al. [6] demonstrated the potential of deep learning for diabetic retinopathy screening, skin lesion classification, and lymph node metastasis detection, respectively. Needless to say, there has been a surge of interest in the potential of CNN among radiology researchers, and several studies have already been published in areas such as lesion detection [7], classification [8], segmentation [9], image reconstruction [10, 11], and natural language processing [12]. Familiarity with this state-of-the-art methodology would help not only researchers who apply CNN to their tasks in radiology and medical imaging, but also clinical radiologists, as deep learning may influence their practice in the near future. This article focuses on the basic concepts of CNN and their application to various radiology tasks, and discusses its challenges and future directions. Other deep learning models, such as recurrent neural networks for sequence models, are beyond the scope of this article.

## Terminology

The following terms are consistently employed throughout this article so as to avoid confusion. A “parameter” in this article stands for a variable that is automatically learned during the training process. A “hyperparameter” refers to a variable that needs to be set before the training process starts. A “kernel” refers to the sets of learnable parameters applied in convolution operations. A “weight” is generally used interchangeably with “parameter”; however, we tried to employ this term when referring to a parameter outside of convolution layers, i.e., a kernel, for example in fully connected layers.

## What is CNN: the big picture (Fig. 1)

CNN is a type of deep learning model for processing data that has a grid pattern, such as images, which is inspired by the organization of animal visual cortex [13, 14] and designed to automatically and adaptively learn spatial hierarchies of features, from low- to high-level patterns. CNN is a mathematical construct that is typically composed of three types of layers (or building blocks): convolution, pooling, and fully connected layers. The first two, convolution and pooling layers, perform feature extraction, whereas the third, a fully connected layer, maps the extracted features into final output, such as classification. A convolution layer plays a key role in CNN, which is composed of a stack of mathematical operations, such as convolution, a specialized type of linear operation. In digital images, pixel values are stored in a two-dimensional (2D) grid, i.e., an array of numbers (Fig. 2), and a small grid of parameters called kernel, an optimizable feature extractor, is applied at each image position, which makes CNNs highly efficient for image processing, since a feature may occur anywhere in the image. As one layer feeds its output into the next layer, extracted features can hierarchically and progressively become more complex. The process of optimizing parameters such as kernels is called training, which is performed so as to minimize the difference between outputs and ground truth labels through an optimization algorithm called backpropagation and gradient descent, among others.

**Fig. 1:**
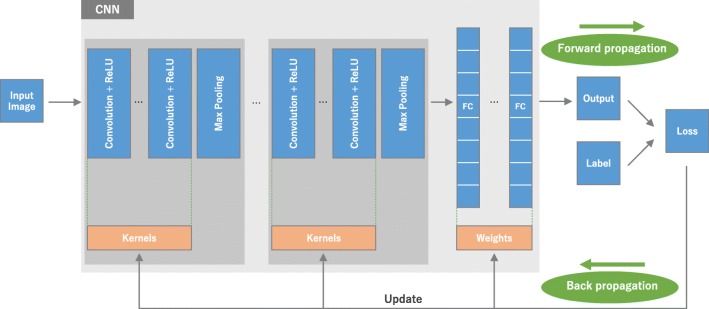
An overview of a convolutional neural network (CNN) architecture and the training process. A CNN is composed of a stacking of several building blocks: convolution layers, pooling layers (e.g., max pooling), and fully connected (FC) layers. A model’s performance under particular kernels and weights is calculated with a loss function through forward propagation on a training dataset, and learnable parameters, i.e., kernels and weights, are updated according to the loss value through backpropagation with gradient descent optimization algorithm. ReLU, rectified linear unit

**Fig. 2:**
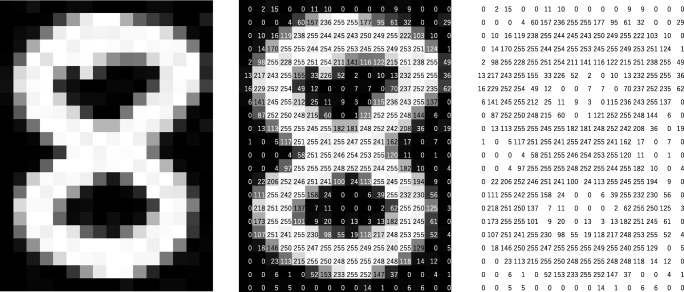
A computer sees an image as an array of numbers. The matrix on the right contains numbers between 0 and 255, each of which corresponds to the pixel brightness in the left image. Both are overlaid in the middle image. The source image was downloaded via http://yann.lecun.com/exdb/mnist

## How is CNN different from other methods employed in radiomics?

Most recent radiomics studies use hand-crafted feature extraction techniques, such as texture analysis, followed by conventional machine learning classifiers, such as random forests and support vector machines [15, 16]. There are several differences to note between such methods and CNN. First, CNN does not require hand-crafted feature extraction. Second, CNN architectures do not necessarily require segmentation of tumors or organs by human experts. Third, CNN is far more data hungry because of its millions of learnable parameters to estimate, and, thus, is more computationally expensive, resulting in requiring graphical processing units (GPUs) for model training.

## Building blocks of CNN architecture

The CNN architecture includes several building blocks, such as convolution layers, pooling layers, and fully connected layers. A typical architecture consists of repetitions of a stack of several convolution layers and a pooling layer, followed by one or more fully connected layers. The step where input data are transformed into output through these layers is called forward propagation (Fig. 1). Although convolution and pooling operations described in this section are for 2D-CNN, similar operations can also be performed for three-dimensional (3D)-CNN.

### Convolution layer

A convolution layer is a fundamental component of the CNN architecture that performs feature extraction, which typically consists of a combination of linear and nonlinear operations, i.e., convolution operation and activation function.

#### Convolution

**Fig. 3:**
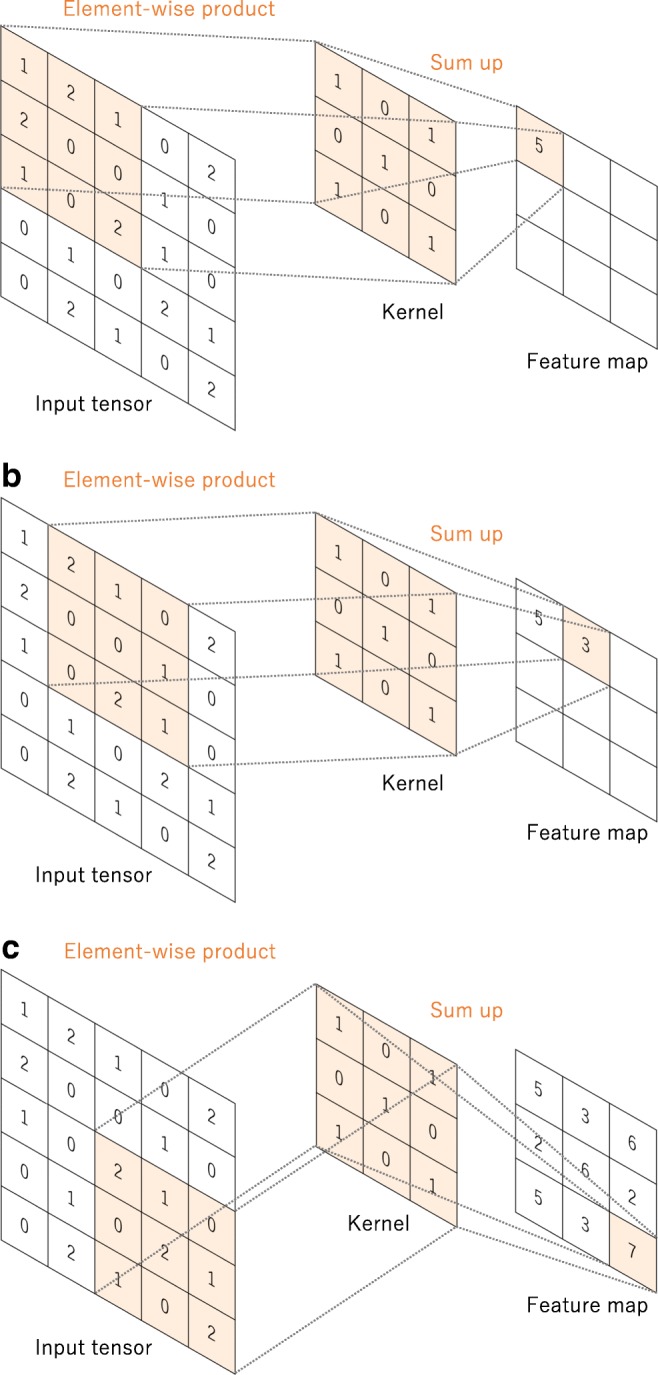

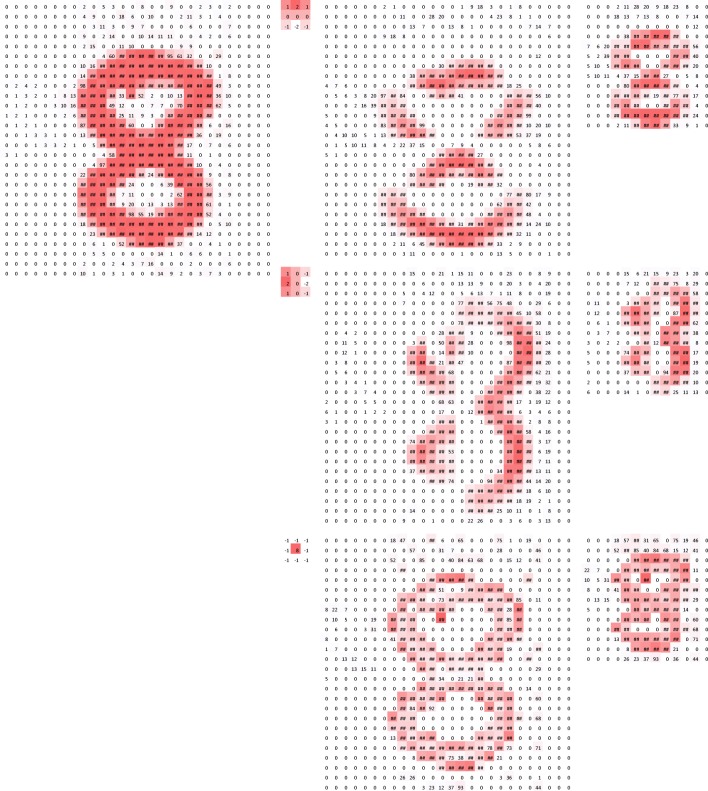
**a**–**c** An example of convolution operation with a kernel size of 3 × 3, no padding, and a stride of 1. A kernel is applied across the input tensor, and an element-wise product between each element of the kernel and the input tensor is calculated at each location and summed to obtain the output value in the corresponding position of the output tensor, called a feature map. **d** Examples of how kernels in convolution layers extract features from an input tensor are shown. Multiple kernels work as different feature extractors, such as a horizontal edge detector (top), a vertical edge detector (middle), and an outline detector (bottom). Note that the left image is an input, those in the middle are kernels, and those in the right are output feature maps

Convolution is a specialized type of linear operation used for feature extraction, where a small array of numbers, called a kernel, is applied across the input, which is an array of numbers, called a tensor. An element-wise product between each element of the kernel and the input tensor is calculated at each location of the tensor and summed to obtain the output value in the corresponding position of the output tensor, called a feature map (Fig. 3a–c). This procedure is repeated applying multiple kernels to form an arbitrary number of feature maps, which represent different characteristics of the input tensors; different kernels can, thus, be considered as different feature extractors (Fig. 3d). Two key hyperparameters that define the convolution operation are size and number of kernels. The former is typically 3 × 3, but sometimes 5 × 5 or 7 × 7. The latter is arbitrary, and determines the depth of output feature maps.

**Fig. 4:**
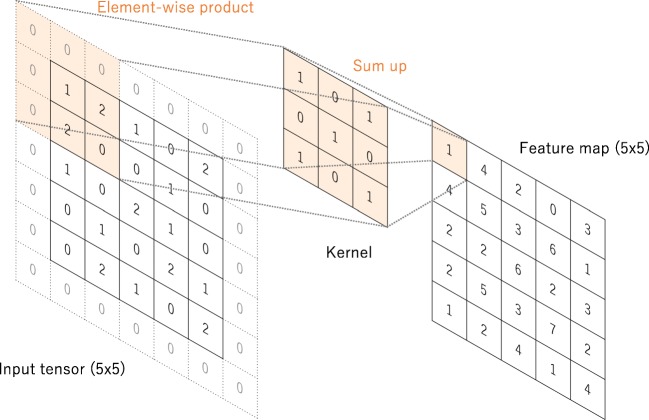
A convolution operation with zero padding so as to retain in-plane dimensions. Note that an input dimension of 5 × 5 is kept in the output feature map. In this example, a kernel size and a stride are set as 3 × 3 and 1, respectively

The convolution operation described above does not allow the center of each kernel to overlap the outermost element of the input tensor, and reduces the height and width of the output feature map compared to the input tensor. Padding, typically zero padding, is a technique to address this issue, where rows and columns of zeros are added on each side of the input tensor, so as to fit the center of a kernel on the outermost element and keep the same in-plane dimension through the convolution operation (Fig. 4). Modern CNN architectures usually employ zero padding to retain in-plane dimensions in order to apply more layers. Without zero padding, each successive feature map would get smaller after the convolution operation.

The distance between two successive kernel positions is called a stride, which also defines the convolution operation. The common choice of a stride is 1; however, a stride larger than 1 is sometimes used in order to achieve downsampling of the feature maps. An alternative technique to perform downsampling is a pooling operation, as described below.

The key feature of a convolution operation is weight sharing: kernels are shared across all the image positions. Weight sharing creates the following characteristics of convolution operations: (1) letting the local feature patterns extracted by kernels translation b invariant as kernels travel across all the image positions and detect learned local patterns, (2) learning spatial hierarchies of feature patterns by downsampling in conjunction with a pooling operation, resulting in capturing an increasingly larger field of view, and (3) increasing model efficiency by reducing the number of parameters to learn in comparison with fully connected neural networks.

As described later, the process of training a CNN model with regard to the convolution layer is to identify the kernels that work best for a given task based on a given training dataset. Kernels are the only parameters automatically learned during the training process in the convolution layer; on the other hand, the size of the kernels, number of kernels, padding, and stride are hyperparameters that need to be set before the training process starts (Table 1).

**Table 1:** A list of parameters and hyperparameters in a convolutional neural network (CNN)

	Parameters	Hyperparameters
Convolution layer	Kernels	Kernel size, number of kernels, stride, padding, activation function
Pooling layer	None	Pooling method, filter size, stride, padding
Fully connected layer	Weights	Number of weights, activation function
Others		Model architecture, optimizer, learning rate, loss function, mini-batch size, epochs, regularization, weight initialization, dataset splitting

Note that a parameter is a variable that is automatically optimized during the training process and a hyperparameter is a variable that needs to be set beforehand

#### Nonlinear activation function

**Fig. 5:**
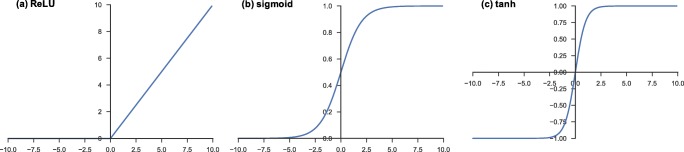
Activation functions commonly applied to neural networks: **a** rectified linear unit (ReLU), **b** sigmoid, and **c** hyperbolic tangent (tanh)

The outputs of a linear operation such as convolution are then passed through a nonlinear activation function. Although smooth nonlinear functions, such as sigmoid or hyperbolic tangent (tanh) function, were used previously because they are mathematical representations of a biological neuron behavior, the most common nonlinear activation function used presently is the rectified linear unit (ReLU), which simply computes the function: f(*x*) = max(0, *x*) (Fig. 5) [1, 3, 17–19].

### Pooling layer

A pooling layer provides a typical downsampling operation which reduces the in-plane dimensionality of the feature maps in order to introduce a translation invariance to small shifts and distortions, and decrease the number of subsequent learnable parameters. It is of note that there is no learnable parameter in any of the pooling layers, whereas filter size, stride, and padding are hyperparameters in pooling operations, similar to convolution operations.

#### Max pooling

**Fig. 6:**
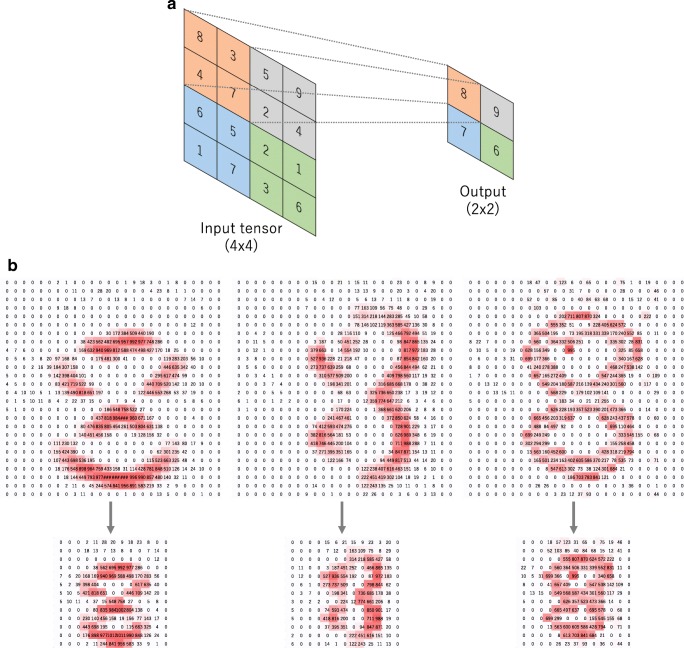
**a** An example of max pooling operation with a filter size of 2 × 2, no padding, and a stride of 2, which extracts 2 × 2 patches from the input tensors, outputs the maximum value in each patch, and discards all the other values, resulting in downsampling the in-plane dimension of an input tensor by a factor of 2. **b** Examples of the max pooling operation on the same images in Fig. 3b. Note that images in the upper row are downsampled by a factor of 2, from 26 × 26 to 13 × 13

The most popular form of pooling operation is max pooling, which extracts patches from the input feature maps, outputs the maximum value in each patch, and discards all the other values (Fig. 6). A max pooling with a filter of size 2 × 2 with a stride of 2 is commonly used in practice. This downsamples the in-plane dimension of feature maps by a factor of 2. Unlike height and width, the depth dimension of feature maps remains unchanged.

#### Global average pooling

Another pooling operation worth noting is a global average pooling [20]. A global average pooling performs an extreme type of downsampling, where a feature map with size of height × width is downsampled into a 1 × 1 array by simply taking the average of all the elements in each feature map, whereas the depth of feature maps is retained. This operation is typically applied only once before the fully connected layers. The advantages of applying global average pooling are as follows: (1) reduces the number of learnable parameters and (2) enables the CNN to accept inputs of variable size.

### Fully connected layer

The output feature maps of the final convolution or pooling layer is typically flattened, i.e., transformed into a one-dimensional (1D) array of numbers (or vector), and connected to one or more fully connected layers, also known as dense layers, in which every input is connected to every output by a learnable weight. Once the features extracted by the convolution layers and downsampled by the pooling layers are created, they are mapped by a subset of fully connected layers to the final outputs of the network, such as the probabilities for each class in classification tasks. The final fully connected layer typically has the same number of output nodes as the number of classes. Each fully connected layer is followed by a nonlinear function, such as ReLU, as described above.

### Last layer activation function

The activation function applied to the last fully connected layer is usually different from the others. An appropriate activation function needs to be selected according to each task. An activation function applied to the multiclass classification task is a softmax function which normalizes output real values from the last fully connected layer to target class probabilities, where each value ranges between 0 and 1 and all values sum to 1. Typical choices of the last layer activation function for various types of tasks are summarized in Table 2.

**Table 2:** A list of commonly applied last layer activation functions for various tasks

Task	Last layer activation function
Binary classification	Sigmoid
Multiclass single-class classification	Softmax
Multiclass multiclass classification	Sigmoid
Regression to continuous values	Identity

## Training a network

Training a network is a process of finding kernels in convolution layers and weights in fully connected layers which minimize differences between output predictions and given ground truth labels on a training dataset. Backpropagation algorithm is the method commonly used for training neural networks where loss function and gradient descent optimization algorithm play essential roles. A model performance under particular kernels and weights is calculated by a loss function through forward propagation on a training dataset, and learnable parameters, namely kernels and weights, are updated according to the loss value through an optimization algorithm called backpropagation and gradient descent, among others (Fig. 1).

### Loss function

A loss function, also referred to as a cost function, measures the compatibility between output predictions of the network through forward propagation and given ground truth labels. Commonly used loss function for multiclass classification is cross entropy, whereas mean squared error is typically applied to regression to continuous values. A type of loss function is one of the hyperparameters and needs to be determined according to the given tasks.

### Gradient descent

**Fig. 7:**
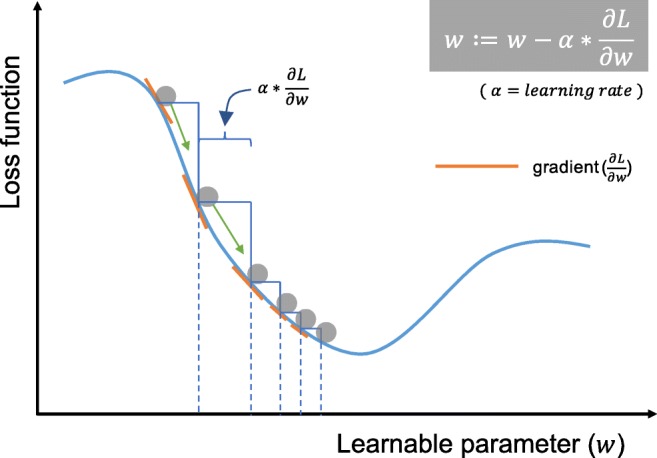
Gradient descent is an optimization algorithm that iteratively updates the learnable parameters so as to minimize the loss, which measures the distance between an output prediction and a ground truth label. The gradient of the loss function provides the direction in which the function has the steepest rate of increase, and all parameters are updated in the negative direction of the gradient with a step size determined based on a learning rate

Gradient descent is commonly used as an optimization algorithm that iteratively updates the learnable parameters, i.e., kernels and weights, of the network so as to minimize the loss. The gradient of the loss function provides us the direction in which the function has the steepest rate of increase, and each learnable parameter is updated in the negative direction of the gradient with an arbitrary step size determined based on a hyperparameter called learning rate (Fig. 7). The gradient is, mathematically, a partial derivative of the loss with respect to each learnable parameter, and a single update of a parameter is formulated as follows:

$$ w:=w-\alpha \ast \frac{\partial L}{\partial w} $$

where *w* stands for each learnable parameter, *α* stands for a learning rate, and *L* stands for a loss function. It is of note that, in practice, a learning rate is one of the most important hyperparameters to be set before the training starts. In practice, for reasons such as memory limitations, the gradients of the loss function with regard to the parameters are computed by using a subset of the training dataset called mini-batch, and applied to the parameter updates. This method is called mini-batch gradient descent, also frequently referred to as stochastic gradient descent (SGD), and a mini-batch size is also a hyperparameter. In addition, many improvements on the gradient descent algorithm have been proposed and widely used, such as SGD with momentum, RMSprop, and Adam [21–23], though the details of these algorithms are beyond the scope of this article.

## Data and ground truth labels

Data and ground truth labels are the most important components in research applying deep learning or other machine learning methods. As a famous proverb originating in computer science notes: “Garbage in, garbage out.” Careful collection of data and ground truth labels with which to train and test a model is mandatory for a successful deep learning project, but obtaining high-quality labeled data can be costly and time-consuming. While there may be multiple medical image datasets open to the public [24, 25], special attention should be paid in these cases to the quality of the ground truth labels.

**Fig. 8:**
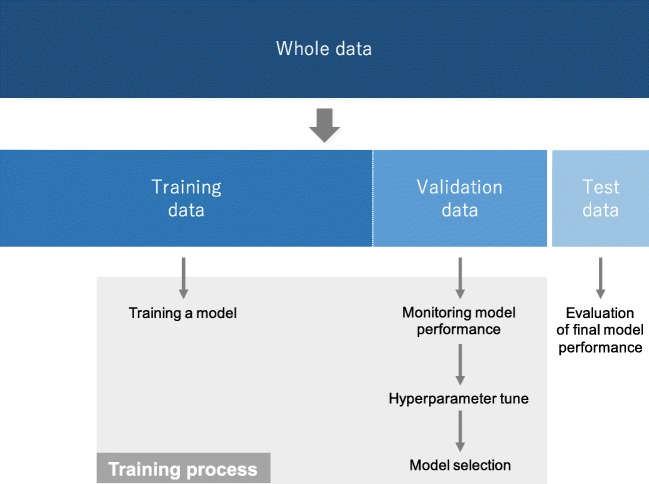
Available data are typically split into three sets: a training, a validation, and a test set. A training set is used to train a network, where loss values are calculated via forward propagation and learnable parameters are updated via backpropagation. A validation set is used to monitor the model performance during the training process, fine-tune hyperparameters, and perform model selection. A test set is ideally used only once at the very end of the project in order to evaluate the performance of the final model that is fine-tuned and selected on the training process with training and validation sets

Available data are typically split into three sets: a training, a validation, and a test set (Fig. 8), though there are some variants, such as cross validation. A training set is used to train a network, where loss values are calculated via forward propagation and learnable parameters are updated via backpropagation. A validation set is used to evaluate the model during the training process, fine-tune hyperparameters, and perform model selection. A test set is ideally used only once at the very end of the project in order to evaluate the performance of the final model that was fine-tuned and selected on the training process with training and validation sets.

Separate validation and test sets are needed because training a model always involves fine-tuning its hyperparameters and performing model selection. As this process is performed based on the performance on the validation set, some information about this validation set leaks into the model itself, i.e., overfitting to the validation set, even though the model is never directly trained on it for the learnable parameters. For that reason, it is guaranteed that the model with fine-tuned hyperparameters on the validation set will perform well on this same validation set. Therefore, a completely unseen dataset, i.e., a separate test set, is necessary for the appropriate evaluation of the model performance, as what we care about is the model performance on never-before-seen data, i.e., generalizability.

It is worthy of mention that the term “validation” is used differently in the medical field and the machine learning field [26]. As described above, in machine learning, the term “validation” usually refers to a step to fine-tune and select models during the training process. On the other hand, in medicine, “validation” usually stands for the process of verifying the performance of a prediction model, which is analogous to the term “test” in machine learning. In order to avoid this confusion, the word “development set” is sometimes used as a substitute for “validation set”.

## Overfitting

**Fig. 9:**
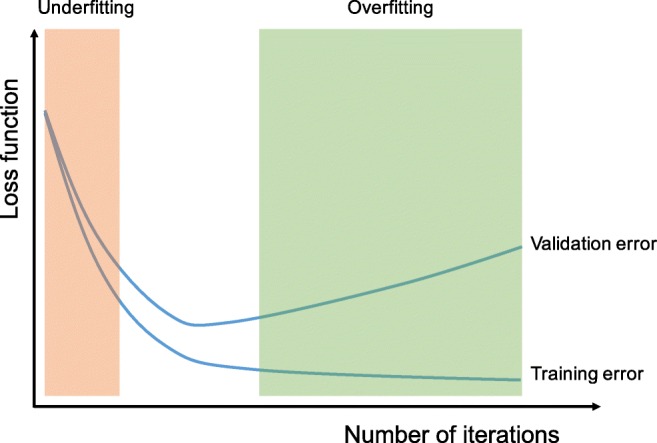
A routine check for recognizing overfitting is to monitor the loss on the training and validation sets during the training iteration. If the model performs well on the training set compared to the validation set, then the model has been overfit to the training data. If the model performs poorly on both training and validation sets, then the model has been underfit to the data. Although the longer a network is trained, the better it performs on the training set, at some point, the network fits too well to the training data and loses its capability to generalize

Overfitting refers to a situation where a model learns statistical regularities specific to the training set, i.e., ends up memorizing the irrelevant noise instead of learning the signal, and, therefore, performs less well on a subsequent new dataset. This is one of the main challenges in machine learning, as an overfitted model is not generalizable to never-seen-before data. In that sense, a test set plays a pivotal role in the proper performance evaluation of machine learning models, as discussed in the previous section. A routine check for recognizing overfitting to the training data is to monitor the loss and accuracy on the training and validation sets (Fig. 9). If the model performs well on the training set compared to the validation set, then the model has likely been overfit to the training data. There have been several methods proposed to minimize overfitting (Table 3). The best solution for reducing overfitting is to obtain more training data. A model trained on a larger dataset typically generalizes better, though that is not always attainable in medical imaging. The other solutions include regularization with dropout or weight decay, batch normalization, and data augmentation, as well as reducing architectural complexity. Dropout is a recently introduced regularization technique where randomly selected activations are set to 0 during the training, so that the model becomes less sensitive to specific weights in the network [27]. Weight decay, also referred to as L2 regularization, reduces overfitting by penalizing the model’s weights so that the weights take only small values. Batch normalization is a type of supplemental layer which adaptively normalizes the input values of the following layer, mitigating the risk of overfitting, as well as improving gradient flow through the network, allowing higher learning rates, and reducing the dependence on initialization [28]. Data augmentation is also effective for the reduction of overfitting, which is a process of modifying the training data through random transformations, such as flipping, translation, cropping, rotating, and random erasing, so that the model will not see exactly the same inputs during the training iterations [29]. In spite of these efforts, there is still a concern of overfitting to the validation set rather than to the training set because of information leakage during the hyperparameter fine-tuning and model selection process. Therefore, reporting the performance of the final model on a separate (unseen) test set, and ideally on external validation datasets if applicable, is crucial for verifying the model generalizability.

**Table 3:** A list of common methods to mitigate overfitting

How to mitigate overfitting	
More training data	
Data augmentation	
Regularization (weight decay, dropout)	
Batch normalization	
Reduce architecture complexity	

## Training on a small dataset

An abundance of well-labeled data in medical imaging is desirable but rarely available due to the cost and necessary workload of radiology experts. There are a couple of techniques available to train a model efficiently on a smaller dataset: data augmentation and transfer learning. As data augmentation was briefly covered in the previous section, this section focuses on transfer learning.

**Fig. 10:**
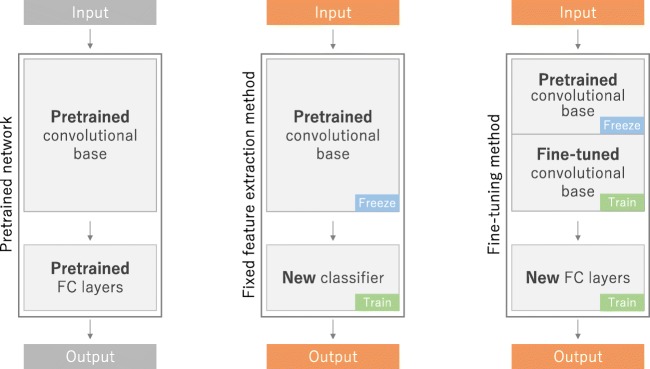
Transfer learning is a common and effective strategy to train a network on a small dataset, where a network is pretrained on an extremely large dataset, such as ImageNet, then reused and applied to the given task of interest. A fixed feature extraction method is a process to remove FC layers from a pretrained network and while maintaining the remaining network, which consists of a series of convolution and pooling layers, referred to as the convolutional base, as a fixed feature extractor. In this scenario, any machine learning classifier, such as random forests and support vector machines, as well as the usual FC layers, can be added on top of the fixed feature extractor, resulting in training limited to the added classifier on a given dataset of interest. A fine-tuning method, which is more often applied to radiology research, is to not only replace FC layers of the pretrained model with a new set of FC layers to retrain them on a given dataset, but to fine-tune all or part of the kernels in the pretrained convolutional base by means of backpropagation. FC, fully connected

Transfer learning is a common and effective strategy to train a network on a small dataset, where a network is pretrained on an extremely large dataset, such as ImageNet, which contains 1.4 million images with 1000 classes, then reused and applied to the given task of interest. The underlying assumption of transfer learning is that generic features learned on a large enough dataset can be shared among seemingly disparate datasets. This portability of learned generic features is a unique advantage of deep learning that makes itself useful in various domain tasks with small datasets. At present, many models pretrained on the ImageNet challenge dataset are open to the public and readily accessible, along with their learned kernels and weights, such as AlexNet [3], VGG [30], ResNet [31], Inception [32], and DenseNet [33]. In practice, there are two ways to utilize a pretrained network: fixed feature extraction and fine-tuning (Fig. 10).

A fixed feature extraction method is a process to remove fully connected layers from a network pretrained on ImageNet and while maintaining the remaining network, which consists of a series of convolution and pooling layers, referred to as the convolutional base, as a fixed feature extractor. In this scenario, any machine learning classifier, such as random forests and support vector machines, as well as the usual fully connected layers in CNNs, can be added on top of the fixed feature extractor, resulting in training limited to the added classifier on a given dataset of interest. This approach is not common in deep learning research on medical images because of the dissimilarity between ImageNet and given medical images.

A fine-tuning method, which is more often applied to radiology research, is to not only replace fully connected layers of the pretrained model with a new set of fully connected layers to retrain on a given dataset, but to fine-tune all or part of the kernels in the pretrained convolutional base by means of backpropagation. All the layers in the convolutional base can be fine-tuned or, alternatively, some earlier layers can be fixed while fine-tuning the rest of the deeper layers. This is motivated by the observation that the early-layer features appear more generic, including features such as edges applicable to a variety of datasets and tasks, whereas later features progressively become more specific to a particular dataset or task [34, 35].

One drawback of transfer learning is its constraints on input dimensions. The input image has to be 2D with three channels relevant to RGB because the ImageNet dataset consists of 2D color images that have three channels (RGB: red, green, and blue), whereas medical grayscale images have only one channel (levels of gray). On the other hand, the height and width of an input image can be arbitrary, but not too small, by adding a global pooling layer between the convolutional base and added fully connected layers.

There has also been increasing interest in taking advantage of unlabeled data, i.e., semi-supervised learning, to overcome a small-data problem. Examples of this attempt include pseudo-label [36] and incorporating generative models, such as generative adversarial networks (GANs) [37]. However, whether these techniques can really help improve the performance of deep learning in radiology is not clear and remains an area of active investigation.

## Applications in radiology

This section introduces recent applications within radiology, which are divided into the following categories: classification, segmentation, detection, and others.

### Classification

**Fig. 11:**
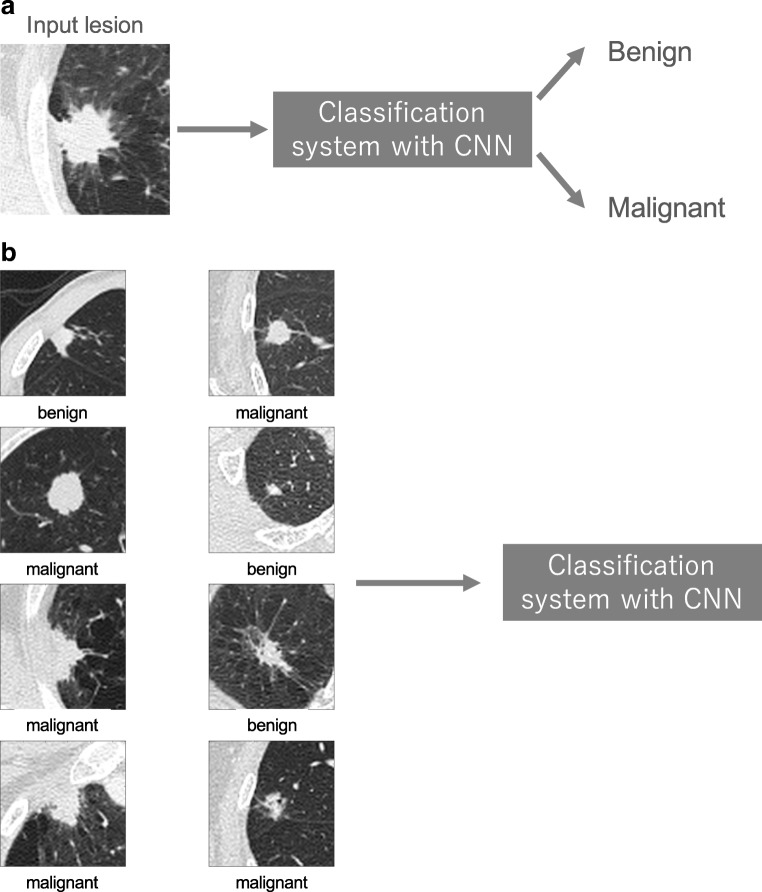

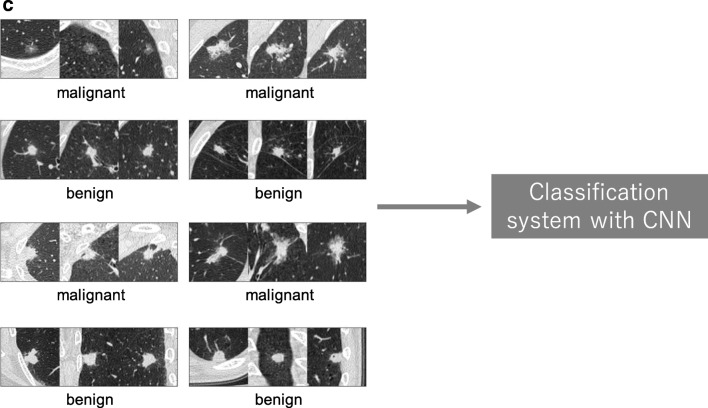
A schematic illustration of a classification system with CNN and representative examples of its training data. **a** Classification system with CNN in the deployment phase. **b**, **c** Training data used in training phase

In medical image analysis, classification with deep learning usually utilizes target lesions depicted in medical images, and these lesions are classified into two or more classes. For example, deep learning is frequently used for the classification of lung nodules on computed tomography (CT) images as benign or malignant (Fig. 11a). As shown, it is necessary to prepare a large number of training data with corresponding labels for efficient classification using CNN. For lung nodule classification, CT images of lung nodules and their labels (i.e., benign or cancerous) are used as training data. Figure 11b, c show two examples of training data of lung nodule classification between benign lung nodule and primary lung cancer; Fig. 11b shows the training data where each datum includes an axial image and its label, and Fig. 11c shows the training data where each datum includes three images (axial, coronal, and sagittal images of a lung nodule) and their labels. After training CNN, the target lesions of medical images can be specified in the deployment phase by medical doctors or computer-aided detection (CADe) systems [38].

Because 2D images are frequently utilized in computer vision, deep learning networks developed for the 2D images (2D-CNN) are not directly applied to 3D images obtained in radiology [thin-slice CT or 3D-magnetic resonance imaging (MRI) images]. To apply deep learning to 3D radiological images, different approaches such as custom architectures are used. For example, Setio et al. [39] used a multistream CNN to classify nodule candidates of chest CT images between nodules or non-nodules in the databases of the Lung Image Database Consortium and Image Database Resource Initiative (LIDC-IDRI) [40], ANODE09 [41], and the Danish Lung Cancer Screening Trial [42]. They extracted differently oriented 2D image patches based on multiplanar reconstruction from one nodule candidate (one or nine patches per candidate), and these patches were used in separate streams and merged in the fully connected layers to obtain the final classification output. One previous study used 3D-CNN for fully capturing the spatial 3D context information of lung nodules [43]. Their 3D-CNN performed binary classification (benign or malignant nodules) and ternary classification (benign lung nodule, and malignant primary and secondary lung cancers) using the LIDC-IDRI database. They used a multiview strategy in 3D-CNN, whose inputs were obtained by cropping three 3D patches of a lung nodule in different sizes and then resizing them into the same size. They also used the 3D Inception model in their 3D-CNN, where the network path was divided into multiple branches with different convolution and pooling operators.

Time series data are frequently obtained in radiological examinations such as dynamic contrast-enhanced CT/MRI or dynamic radio isotope (RI)/positron emission tomography (PET). One previous study used CT image sets of liver masses over three phases (non-enhanced CT, and enhanced CT in arterial and delayed phases) for the classification of liver masses with 2D-CNN [8]. To utilize time series data, the study used triphasic CT images as 2D images with three channels, which corresponds to the RGB color channels in computer vision, for 2D-CNN. The study showed that 2D-CNN using triphasic CT images was superior to that using biphasic or monophasic CT images.

### Segmentation

**Fig. 12:**
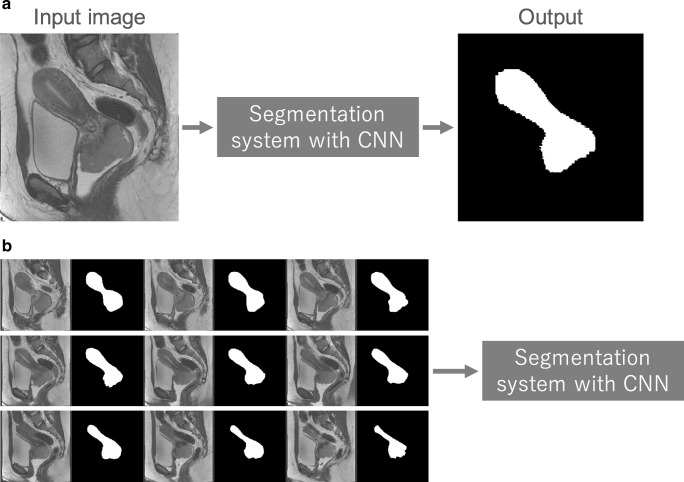
A schematic illustration of the system for segmenting a uterus with a malignant tumor and representative examples of its training data. **a** Segmentation system with CNN in deployment phase. **b** Training data used in the training phase. Note that original images and corresponding manual segmentations are arranged next to each other

Segmentation of organs or anatomical structures is a fundamental image processing technique for medical image analysis, such as quantitative evaluation of clinical parameters (organ volume and shape) and computer-aided diagnosis (CAD) system. In the previous section, classification depends on the segmentation of lesions of interest. Segmentation can be performed manually by radiologists or dedicated personnel, a time-consuming process. However, one can also apply CNN to this task as well. Figure 12a shows a representative example of segmentation of the uterus with a malignant tumor on MRI [24, 44, 45]. In most cases, a segmentation system directly receives an entire image and outputs its segmentation result. Training data for the segmentation system consist of the medical images containing the organ or structure of interest and the segmentation result; the latter is mainly obtained from previously performed manual segmentation. Figure 12b shows a representative example of training data for the segmentation system of a uterus with a malignant tumor. In contrast to classification, because an entire image is inputted to the segmentation system, it is necessary for the system to capture the global spatial context of the entire image for efficient segmentation.

One way to perform segmentation is to use a CNN classifier for calculating the probability of an organ or anatomical structure. In this approach, the segmentation process is divided into two steps; the first step is construction of the probability map of the organ or anatomical structure using CNN and image patches, and the second is a refinement step where the global context of images and the probability map are utilized. One previous study used a 3D-CNN classifier for segmentation of the liver on 3D CT images [46]. The input of 3D-CNN were 3D image patches collected from entire 3D CT images, and the 3D-CNN calculated probabilities for the liver from the image patches. By calculating the probabilities of the liver being present for each image patch, a 3D probability map of the liver was obtained. Then, an algorithm called graph cut [47] was used for refinement of liver segmentation, based on the probability map of the liver. In this method, the local context of CT images was evaluated by 3D-CNN and the global context was evaluated by the graph cut algorithm.

Although segmentation based on image patch was successfully performed in deep learning, U-net of Ronneberger et al. [48] outperformed the image patch-based method on the ISBI [IEEE (The Institute of Electrical and Electronics Engineers) International Symposium on Biomedical Imaging] challenge for segmentation of neuronal structures in electron microscopic images. The architecture of U-net consists of a contracting path to capture anatomical context and a symmetric expanding path that enables precise localization. Although it was difficult to capture global context and local context at the same time by using the image patch-based method, U-net enabled the segmentation process to incorporate a multiscale spatial context. As a result, U-net could be trained end-to-end from a limited number of training data.

One potential approach of using U-net in radiology is to extend U-net for 3D radiological images, as shown in classification. For example, V-net was suggested as an extension of U-net for segmentation of the prostate on volumetric MRI images [49]. In the study, V-net utilized a loss function based on the Dice coefficient between segmentation results and ground truth, which directly reflected the quality of prostate segmentation. Another study [9] utilized two types of 3D U-net for segmenting liver and liver mass on 3D CT images, which was named cascaded fully convolutional neural networks; one type of U-net was used for segmentation of the liver and the other type for the segmentation of liver mass using the liver segmentation results. Because the second type of 3D U-net focused on the segmentation of liver mass, the segmentation of liver mass was more efficiently performed than single 3D U-net.

### Detection

A common task for radiologists is to detect abnormalities within medical images. Abnormalities can be rare and they must be detected among many normal cases. One previous study investigated the usefulness of 2D-CNN for detecting tuberculosis on chest radiographs [7]. The study utilized two different types of 2D-CNN, AlexNet [3] and GoogLeNet [32], to detect pulmonary tuberculosis on chest radiographs. To develop the detection system and evaluate its performance, the dataset of 1007 chest radiographs was used. According to the results, the best area under the curve of receiver operating characteristic curves for detecting pulmonary tuberculosis from healthy cases was 0.99, which was obtained by ensemble of the AlexNet and GoogLeNet 2D-CNNs.

Nearly 40 million mammography examinations are performed in the USA every year. These examinations are mainly performed for screening programs aimed at detecting breast cancer at an early stage. A comparison between a CNN-based CADe system and a reference CADe system relying on hand-crafted imaging features was performed previously [50]. Both systems were trained on a large dataset of around 45,000 images. The two systems shared the candidate detection system. The CNN-based CADe system classified the candidate based on its region of interest, and the reference CADe system classified it based on the hand-crafted imaging features obtained from the results of a traditional segmentation algorithm. The results show that the CNN-based CADe system outperformed the reference CADe system at low sensitivity and achieved comparable performance at high sensitivity.

### Others

**Fig. 13:**
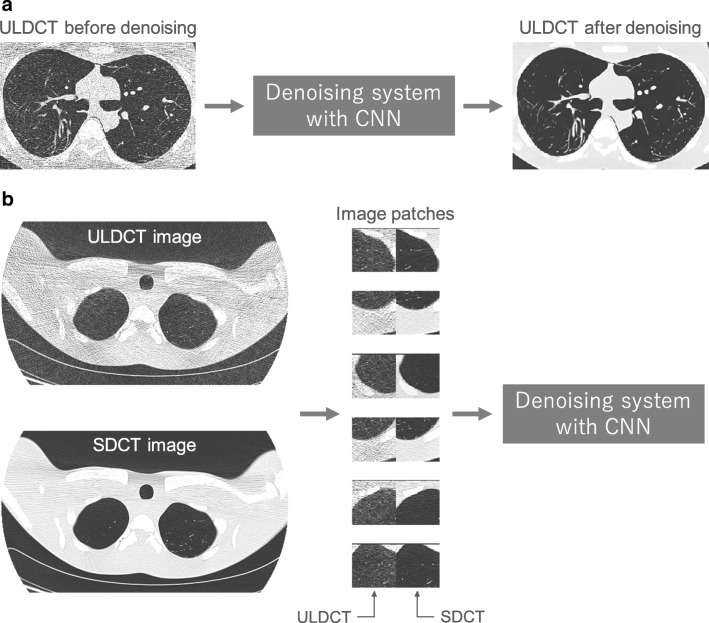
A schematic illustration of the system for denoising an ultra-low-dose CT (ULDCT) image of phantom and representative examples of its training data. **a** Denoising system with CNN in deployment phase. **b** Training data used in training phase. SDCT, standard-dose CT

Low-dose CT has been increasingly used in clinical situations. For example, low-dose CT was shown to be useful for lung cancer screening [51]. Because noisy images of low-dose CT hindered the reliable evaluation of CT images, many techniques of image processing were used for denoising low-dose CT images. Two previous studies showed that low-dose and ultra-low-dose CT images could be effectively denoised using deep learning [52, 53]. Their systems divided the noisy CT image into image patches, denoised the image patches, then reconstructed a new CT image from the denoised image patches. Deep learning with encoder–decoder architecture was used for their systems to denoise image patches. Training data for the denoising systems consisted of pairs of image patches, which are obtained from standard-dose CT and low-dose CT. Figure 13 shows a representative example of the training data of the systems.

One previous study [54] used U-net to solve the inverse problem in imaging for obtaining a noiseless CT image reconstructed from a subsampled sinogram (projection data). To train U-net for reconstructing a noiseless CT image from the subsampled sinogram, the training data of U-net consist of (i) noisy CT images obtained from subsampled sinogram by filtered backprojection (FBP) and (ii) noiseless CT images obtained from the original sinogram. The study suggested that, while it would be possible to train U-net for reconstructing CT images directly from the sinogram, performing the FBP first greatly simplified the training. As a refinement of the original U-net, the study added a skip connection between the input and output for residual learning. Their study showed that U-net could effectively produce noiseless CT images from the subsampled sinogram.

Although deep learning requires a large number of training data, building such a large-scale training data of radiological images is a challenging problem. One main challenge is the cost of annotation (labeling); the annotation cost for a radiological image is much larger than a general image because radiologist expertise is required for annotation. To tackle this problem, one previous study [55] utilized radiologists’ annotations which are routinely added to radiologists’ reports (such as circle, arrow, and square). The study obtained 33,688 bounding boxes of lesions from the annotation of radiologists’ reports. Then, unsupervised lesion categorization was performed to speculate labels of the lesions in the bounding box. To perform unsupervised categorization, the following three steps were iteratively performed: (i) feature extraction using pretrained VGG16 model [30] from the lesions in the bounding box, (ii) clustering of the features, and (iii) fine-tuning of VGG16 based on the results of the clustering. The study named the labels obtained from the results of clustering as pseudo-category labels. The study also suggested that the detection system was built using the Faster R-CNN method [56], the lesions in the bounding box, and their corresponding pseudo-category. The results demonstrate that detection accuracy could be significantly improved by incorporating pseudo-category labels.

Radiologists routinely produce their reports as results of interpreting medical images. Because they summarize the medical images as text data in the reports, it might be possible to collect useful information about disease diagnosis effectively by analyzing the radiologists’ reports. One previous study [12] evaluated the performance of a CNN model, compared with a traditional natural language processing model, in extracting pulmonary embolism findings from chest CT. By using word embedding, words in the radiological reports can be converted to meaningful vectors [57]. For example, the following equation holds by using vector representation with word embedding: king – man + woman = queen. In the previous study, word embedding enabled the radiological reports to be converted to a matrix (or image) of size 300 × 300. By using this representation, 2D-CNN could be used to classify the reports as pulmonary embolism or not. Their results showed that the performance of the CNN model was equivalent to or beyond that of the traditional model.

## Challenges and future directions

Although the recent advancements of deep learning have been astonishing, there still exist challenges to its application to medical imaging.

**Fig. 14:**
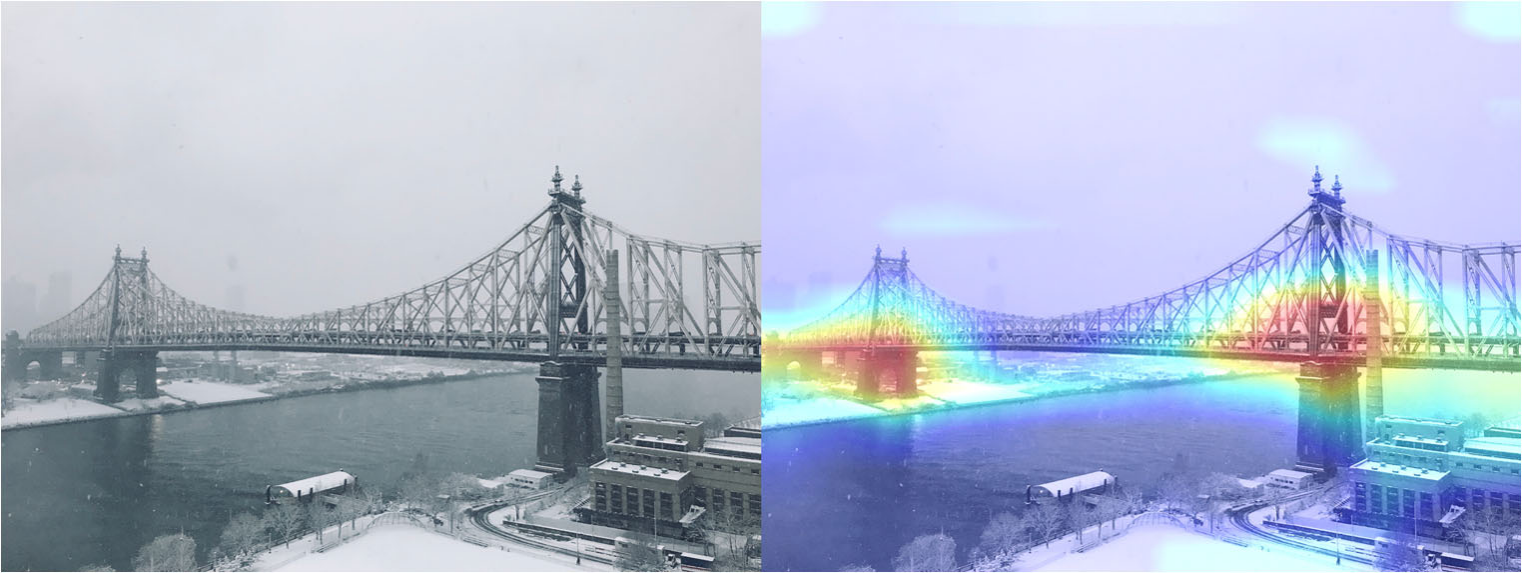
An example of a class activation map (CAM) [58]. A CNN network trained on ImageNet classified the left image as a “bridge pier”. A heatmap for the category of “bridge pier”, generated by a method called Grad-CAM [59], is superimposed (right image), which indicates the discriminative image regions used by the CNN for the classification

**Fig. 15:**
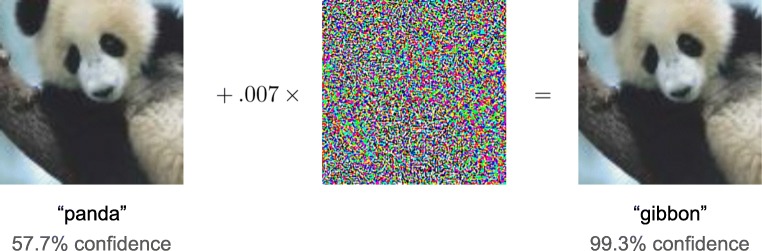
An adversarial example demonstrated by Goodfellow et al. [61]. A network classified the object in the left image as a “panda” with 57.7% confidence. By adding a very small amount of carefully constructed noise (middle image), the network misclassified the object as a “gibbon” with 99.3% confidence on the right image without a visible change to a human. Reprinted with permission from “Explaining and harnessing adversarial examples” by Goodfellow et al. [61]

Deep learning is considered as a black box, as it does not leave an audit trail to explain its decisions. Researchers have proposed several techniques in response to this problem that give insight into what features are identified in the feature maps, called feature visualization, and what part of an input is responsible for the corresponding prediction, called attribution. For feature visualization, Zeiler and Fergus [34] described a way to visualize the feature maps, where the first layers identify small local patterns, such as edges or circles, and subsequent layers progressively combine them into more meaningful structures. For attribution, Zhou et al. proposed a way to produce coarse localization maps, called class activation maps (CAMs), that localize the important regions in an input used for the prediction (Fig. 14) [58, 59]. On the other hand, it is worth noting that researchers have recently that noticed deep neural networks are vulnerable to adversarial examples, which are carefully chosen inputs that cause the network to change output without a visible change to a human (Fig. 15) [60–63]. Although the impact of adversarial examples in the medical domain is unknown, these studies indicate that the way artificial networks see and predict is different from the way we do. Research on the vulnerability of deep neural networks in medical imaging is crucial because the clinical application of deep learning needs extreme robustness for the eventual use in patients, compared to relatively trivial non-medical tasks, such as distinguishing cats or dogs.

Although there are several methods that facilitate learning on smaller datasets as described above, well-annotated large medical datasets are still needed since most of the notable accomplishments of deep learning are typically based on very large amounts of data. Unfortunately, building such datasets in medicine is costly and demands an enormous workload by experts, and may also possess ethical and privacy issues. The goal of large medical datasets is the potential to enhance generalizability and minimize overfitting, as discussed previously. In addition, dedicated medical pretrained networks can probably be proposed once such datasets become available, which may foster deep learning research on medical imaging, though whether transfer learning with such networks improves the performance in the medical field compared to that with ImageNet pretrained models is not clear and remains an area of further investigation.

## Conclusion

Convolutional neural networks (CNNs) have accomplished astonishing achievements across a variety of domains, including medical research, and an increasing interest has emerged in radiology. Although deep learning has become a dominant method in a variety of complex tasks such as image classification and object detection, it is not a panacea. Being familiar with key concepts and advantages of CNN as well as limitations of deep learning is essential in order to leverage it in radiology research with the goal of improving radiologist performance and, eventually, patient care.

## Abbreviations

1D: One-dimensional
2D: Two-dimensional
3D: Three-dimensional
CAD: Computer-aided diagnosis
CADe: Computer-aided detection
CAM: Class activation map
CNN: Convolutional neural network
CT: Computed tomography
FBP: Filtered backprojection
GAN: Generative adversarial network
GPU: Graphical processing unit
IEEE: The Institute of Electrical and Electronics Engineers
ILSVRC: ImageNet Large Scale Visual Recognition Competition
ISBI: IEEE International Symposium on Biomedical Imaging
LIDC-IDRI: Lung Image Database Consortium and Image Database Resource Initiative
MRI: Magnetic resonance imaging
PET: Positron emission tomography
ReLU: Rectified linear unit
RI: Radio isotope
RGB: Red, green, and blue
SDG: Stochastic gradient descent
